# Thymosin Alpha-1 Has no Beneficial Effect on Restoring CD4+ and CD8+ T Lymphocyte Counts in COVID-19 Patients

**DOI:** 10.3389/fimmu.2021.568789

**Published:** 2021-06-03

**Authors:** Zhenyan Wang, Jun Chen, Cuiyun Zhu, Li Liu, Tangkai Qi, Yinzhong Shen, Yuyi Zhang, Lie Xu, Tao Li, Zhiping Qian, Corklin R. Steinhart, Hongzhou Lu

**Affiliations:** ^1^ Department of Infection and Immunity, Shanghai Public Health Clinical Center, Fudan University, Shanghai, China; ^2^ Medical Examination Center, Shanghai Public Health Clinical Center, Fudan University, Shanghai, China; ^3^ Department of Medical Administration, Shanghai Public Health Clinical Center, Fudan University, Shanghai, China; ^4^ Department of Liver Intensive Care Unit, Shanghai Public Health Clinical Center, Fudan University, Shanghai, China; ^5^ Department of Tuberculosis, Shanghai Public Health Clinical Center, Fudan University, Shanghai, China; ^6^ CAN Community Health, Sarasota, FL, United States; ^7^ The University of Central Florida College of Medicine, Orlando, FL, United States

**Keywords:** COVID-19, thymosin alpha-1, T lymphocyte, CD4, CD8

## Abstract

Dysregulation of immune response was observed in COVID-19 patients. Thymosin alpha 1 (Tα1) is used in the management of COVID-19, because it is known to restore the homeostasis of the immune system during infections and cancers. We aim to observe the longitudinal changes in T lymphocyte subsets and to evaluate the efficacy of Tα1 for COVID-19. A retrospective study was conducted in 275 COVID-19 patients admitted to Shanghai public health clinical center. The clinical and laboratory characteristics between patients with different T lymphocyte phenotypes and those who were and were not treated with Tα1 were compared. Among the 275 patients, 137 (49.8%) were males, and the median age was 51 years [interquartile range (IQR): 37-64]. A total of 126 patients received Tα1 therapy and 149 patients did not. There were 158 (57.5%) patients with normal baseline CD4 counts (median:631/μL, IQR: 501~762) and 117 patients (42.5%) with decreased baseline CD4 counts (median:271/μL, IQR: 201~335). In those with decreased baseline CD4 counts, more patients were older (p<0.001), presented as critically ill (p=0.032) and had hypertension (p=0.008) compared with those with normal CD4 counts. There was no statistical difference in the duration of virus shedding in the upper respiratory tract between the two groups (p=0.214). In both the normal (14 *vs* 11, p=0.028) and the decreased baseline CD4 counts group (15 *vs* 11, p=0.008), duration of virus clearance in the patients with Tα1 therapy was significantly longer than that in those without Tα1 therapy. There was no significant difference in the increase of CD4+ (286 *vs* 326, p=0.851) and CD8+ T cell (154 *vs* 170, p=0.842) counts in the recovery period between the two groups with or without Tα1 therapy. Multivariate linear regression analysis showed that severity of illness (p<0.001) and Tα1 therapy (p=0.001) were associated with virus clearance. In conclusion, reduction of CD4+ T and CD8+ T cell counts were observed in COVID-19 patients. Tα1 may have no benefit on restoring CD4+ and CD8+ T cell counts or on the virus clearance. The use of Tα1 for COVID-19 need to be more fully investigated.

## Introduction

The coronavirus disease 2019 (COVID-19) caused by SARS-CoV-2 emerged in Wuhan China in December 2019, and rapidly spread worldwide. By August 16, 2020, there were nearly 21.3 million confirmed cases and 76,1779 deaths (1). Although there are multiple vaccines that have been developed and are available in many areas (2–4), the number of new confirmed COVID-19 cases and new death globally remains to be increasing. However, there are still no effective antiviral drugs to treat the disease. Some recommended antiviral drugs for COVID-19, such as lopinavir/ritonavir, hydroxychloroquine and remdesivir were proved to have no efficacy (5–9). Therefore, the treatment of COVID-19 is mainly symptomatic and supportive.

The immune response of the host has an important effect on the development and prognosis of infectious diseases. Studies showed that total lymphocytes, CD3+T cells, CD4+ T cells and CD8+ T cells decreased in COVID-19 patients, and significantly decreased T cell lymphocyte subset counts were related to the severity and prognosis of COVID-19 (10–13). In addition to the reduction of T cell counts, the function of the surviving T cells also appeared exhausted in COVID-19 patients (14). Therefore, activation and expansion of innate and adaptive lymphocytes play a major role in COVID-19 (15). Studies have shown that the viral load of SARS-CoV-2 detected from patient respiratory tracts is related to lung injury and disease severity (16). Lower CD4 counts before treatment has predicted a longer duration of virus RNA detection in the upper respiratory tract (17). Thymosin alpha-1 (Tα1) is one of the main active components of thymic hormone, which is composed of 28 amino acid residues. Synthetic preparations of Tα1 have been approved for clinical applications as immunomodulatory biological response modifiers. Tα1 can induce the differentiation and development of T cells, help to enhance the T cell response to antigens, and increases the level of lymphokine receptors on T cells. Tα1 has shown efficacy in the treatment of several diseases in which the immune system is compromised or malfunctioning (18, 19). Dysregulation of immune response was observed in patients with COVID-19. A powerful cytokine storm accompanies COVID-19 pneumonia. Both helper T (Th) cells and suppressor T cells in patients with COVID-19 were below normal levels. The percentage of naive Th cells increased, and memory Th cells decreased in severe cases (20, 21). Based on the above findings, Tα1was used in the treatment of COVID-19. The purpose of this study was to investigate the characteristics of longitudinal alterations of T lymphocyte subsets in COVID-19 patients and the efficacy of Tα1 for the treatment of COVID-19.

## Materials and Methods

### Study Design and Participants

This study enrolled all the patients with COVID-19 who were admitted to the Shanghai Public Health Clinical Center (Shanghai, China) from Jan 20^th^, 2020 to March 16^th^, 2020 (n=275). All patients were diagnosed with COVID-19 according to the Chinese guidelines for diagnosis and treatment of COVID-19 (5). The demographic, clinical and laboratory data of the 275 patients were retrospectively collected, and included gender, age, severity of disease, treatment regimen, comorbidities, CD4+ and CD8+ T cell levels on admission and before discharge, levels of white blood cell, high-sensitivity C-reactive protein, erythrocyte sedimentation rate and lactic dehydrogenase, and duration of virus shedding in the upper respiratory tract.

According to whether they received Tα1 treatment, the patients were divided into two groups: Tα1 therapy group (n=126) and non-Tα1 therapy group (n=149). Decisions regarding whether to use Tα1 therapy was made based on whether the disease was progressing. Tα1 was administered subcutaneously at a dose of 1.6mg twice a week according to the drug instructions until improvement of disease. To identify SARS-CoV-2 infection and persistence, throat swab samples were obtained from all patients each day during hospitalization and tested SARS-CoV-2 RNA using real-time reverse transcriptase polymerase chain reaction assays.

The patients were divided into 2 groups according to their baseline CD4+ T cell counts after obtained at admission: those with abnormal baseline CD4+T cell counts and those with not decreased CD4+ T cell on admission. For each CD4+ T cell group, patients were further divided into subgroups who were either given Tα1 therapy or not. The clinical and laboratory data between the groups and subgroups were compared. The changes in the levels of CD4+ and CD8+T cells throughout the course of COVID-19 were also analyzed.

### Laboratory Test

#### RT-PCR Detection for SARS-CoV-2

The total nucleic acids were extracted from the nasopharyngeal swabs of patients. The open reading frame 1ab (ORF1ab) and nucleocapsid (N) gene were detected by performing real-time PCR assay [DA0992-Detection Kit for 2019-nCoV (PCR-Fluorescence), ABI7500]. The number of cycles (CT value) was used to measure the viral load. A CT value ≤30 was defined as SARS-CoV-2 viral positive. The limit of detection was 500 copies/ml.

#### Detection for T Lymphocyte Subset

CD3+/CD4+/CD8+/CD45+ T-cell counts (cells/μL) were measured by multiple-color flow cytometry with human monoclonal anti-CD3-fluorescein isothiocyanate (FITC), anti-CD8-phycoerythrin (PE), anti-CD45-Peridinin-Chlorophyll-Protein Complex (PerCP), anti-CD4-allophycocyanin (APC) antibodies (BD Multitest™ CD3/CD8/CD45/CD4) according to the manufacturer’s instructions. Fow cytometry was performed using a BD FACS Canto II flow cytometry system (BD Biosciences).

The laboratory reference intervals were determined as follows: CD4 count: 410- 1590 cells/μL, CD8 count:190-1140 cells/μL, LDH:109-245 U/L, hs-CRP 0~10 mg/L, WBC:(3.5-9.5) ×10^9^/L, and lymphocyte: (1.1-3.2) ×10^9^/L, according to instructions of test kits and previously published data (22, 23).

### Statistical Analysis

Data were analyzed using SPSS 24.0 software (IBM Corp., Armonk, NY). Quantitative data with normal distribution were expressed as means ± standard deviation (x ± SD) and compared using t tests; quantitative data with skewed distribution were expressed as median (inter-quartile range, IQR) and compared using the Mann-Whitney U test. Categorical variables were expressed as frequencies and percentages and compared using the chi-square (x2) test or Fisher exact test. The multivariate linear regression analysis was used to identify risk factors associated with duration of virus shedding in the upper respiratory tract. All tests were two tailed, and p<0.05 were considered statistically significant.

## Results

### Demographic and Clinical Characteristics of COVID-19 Patients

A total of 275 confirmed COVID-19 patients were included in this study. Among them, 137 (49.8%) were male and 138 (50.2%) were female, with a median age of 51 years (IQR:37˜64). There were 158 (57.5%) and 117 (42.5%) patients with normal (M:631 cells/μL, IQR: 501˜762) and abnormal (M:271 cells/μL, IQR: 201˜335) baseline CD4+T cell counts, respectively. More patients aged≥60 years were classified as severe or critical type of COVID-19 (p=0.001), and had lower baseline CD4 and CD8 counts (p<0.001), compared with those aged 60 years. Compared with the normal CD4 group, more patients in the abnormal CD4 group were older (p<0.001), had a higher proportion of hypertension (p=0.008), lower CD8+ T cell counts (p<0.001), lactate dehydrogenase (LDH) elevation (p<0.001), received treatment with glucocorticoid (p<0.001), intravenous immunoglobulin (IVIG) (p<0.001) and Tα1 (p=0.001). However, there was no significant difference in the duration of virus shedding in the upper respiratory tract between the two groups (p=0.214). See **Table 1**.

**Table 1:** Demographic and clinical characteristics of patients with COVID-19.

Characteristics	Total (n=275)	Age ≥ 60 years (n=92)	Age < 60 years (n=183)	*p*	With normal baseline CD4 counts (n=158)	With abnormal baseline CD4 counts (n=117)	*P*
Sex, (n, %)							
Male	137 (49.8)	41 (44.6)	96 (52.5)	0.217	74 (46.8)	63 (53.8)	0.250
Age, M (IQR), years	51 (37-64)	66 (64-70)	40 (32-51)	<0.001	45 (33-61)	55 (40-65)	<0.001
≥60, (n, %)	92 (33.5)	92 (100)	0 (0)	<0.001	43 (27.2)	49 (41.9)	0.011
Severity of disease, (n, %)							
mild or moderate	257 (93.5)	79 (85.9)	178 (97.3)	0.001	152 (96.2)	105 (89.7)	0.032
severe or critical	18 (6.5)	13 (14.1)	5 (2.7)		6 (3.8)	12 (10.3)	
Use of IVIG, (n, %)	50 (18.2)	23 (25.0)	27 (14.8)	0.038	12 (7.6)	38 (32.5)	<0.001
Use of Tα1, (n, %)	126 (45.8)	54 (58.7)	72 (39.3)	0.001	59 (37.3)	67 (57.3)	0.001
Baseline CD4 counts, M (IQR), cells/μL	448 (300-654)	391 (207-578)	497 (317-700)	<0.001	631 (501-762)	271 (201-335)	<0.001
Baseline CD8 counts, M (IQR), cells/μL	259 (163-389)	180 (121-262)	303 (195-449)	<0.001	337 (226-464)	170 (119-257)	<0.001
Hospital days, M (IQR)	16 (12-23)	17 (13-25)	16 (12-22)	0.046	16 (12-21)	17 (12-24)	0.361
Comorbidities, (n, %)							
hypertension	57 (22.2)	15 (16.3)	42 (23.0)	0.200	24 (15.2)	33 (28.2)	0.008
diabetes	24 (9.3)	6 (6.5)	18 (9.8)	0.358	10 (6.3)	14 (12)	0.102
coronary heart disease	12 (4.7)	4 (4.3)	8 (4.4)	0.993	4 (2.5)	8 (6.8)	0.084
COPD	4 (1.6)	1 (1.1)	3 (1.6)	0.718	1 (0.6)	3 (2.6)	0.186
WBC (×10^9^/L), M (IQR)	4.77 (3.89-5.94)	5.11 (4.09-6.13)	4.64 (3.74-5.83)	0.076	4.75 (3.79-5.91)	4.85 (4.02-6.14)	0.235
Lymphocytes (×10^9^/L), M (IQR)	1.13 (0.79-1.49)	1.34 (1.06-1.79)	1.01 (0.75-1.38)	<0.001	1.26 (0.93-1.61)	0.96 (0.73-1.33)	<0.001
hs-CRP>10mg/L, n (%)	128 (49.8)	42 (45.7)	104 (56.8)	0.080	77 (48.7)	69 (59.0)	0.092
LDH (U/L), M (IQR)	229 (193-290)	214 (189-252)	241 (198-313)	0.001	214 (188-257)	255 (207-335)	<0.001
Duration of virus shedding in the URT, days, M (IQR)	12 (8~19)	13 (9-21)	12 (8-18)	0.192	12 (8-18)	13 (9-21)	0.214

IVIG, intravenous immunoglobulin; Tα1, thymosin alpha-1; WBC, white blood cell; hs-CRP, high-sensitivity C-reactive protein; LDH, lactic dehydrogenase; URT, upper respiratory tract. The laboratory reference intervals were determined as follows: CD4 count: 410-1590cells/μL, CD8 count:190-1140cells/μL, LDH:109-245U/L, hs-CRP 0~10mg/L, WBC:(3.5~9.5) ×109/L, and lymphocyte: (1.1~3.2) ×109/L, according to instructions of test kits and previously published data (22, 23).

### Effects of Tα1 on the Duration of SARS-CoV-2 Shedding in the Upper Respiratory Tract

Among the 275 COVID-19 patients, 126 patients received Tα1 therapy, with a median number of 5 injections (IQR: 3-7, range: 1-24). Irrespective of baseline CD4+ T cell counts, the duration of SARS-CoV-2 shedding in the upper respiratory tract in the Tα1 subgroup was significantly longer than that in the untreated subgroup [Tα1 vs non-Tα1: 14 vs 11 (p=0.028) in the normal CD4 group, 15 *vs* 11 (p=0.008) in the abnormal CD4 group]. And it is important to note that a higher proportion of patients in the Tα1 group also received glucocorticoid (p < 0.001) and IVIG (p < 0.001) than did the group that did not receive Tα1. See **Table 2**.

**Table 2:** Characteristics of COVID-19 patients with and without therapy with Tα1.

Characteristics	With normal baseline CD4 counts (n=158)		*P*	With decreased baseline CD4 counts (n=117)		*P*	Total (n=275)
	Tα1+ (n=59)	Tα1- (n=99)		Tα1+ (n=67)	Tα1- (n=50)		
Age, M(IQR), years	51 (34-63)	42 (33-60)	0.306	63 (50-68)	47(36-60)	<0.001	51 (37-64)
Sex (n, %)							
male	35 (59.3)	39 (39.4)	0.015	38 (56.7)	25 (50)	0.471	137 (49.8)
Duration of virus shedding in the URT, days, M(IQR)	14 (8-25)	11 (7-17)	0.028	15 (10-22)	11 (8-16)	0.008	12 (8~19)
Use of IVIG (n, %)	12 (20.3)	0	<0.001	32 (47.8)	6 (12.0)	<0.001	50 (18.2)
Use of glucocorticoid (n, %)	10 (16.9)	3 (3.0)	0.005	31 (46.3)	6 (12.0)	<0.001	50 (18.2)
Severity (n, %)							
Mild or moderate	54 (91.5)	98 (99.0)	0.052	55 (82.1)	50 (100)	0.002	257 (93.5)
Severe or critical	5 (8.5)	1 (1.0)		12 (17.9)	0(0)	18(6.5)
Baseline CD4 counts, cells/μL, M(IQR)	637 (498-741)	631 (502-768)	0.723	261 (182-332)	298 (234-341)	0.059	448 (300-654)
Baseline CD8 counts cells/μL, M(IQR)	325 (200-432)	364 (248-494)	0.156	159 (96-229)	211 (134-294)	0.014	259 (163-389)
Hospital days, M(IQR)	18 (14-29)	15 (11-20)	<0.001	21 (15-26)	14 (11-18)	<0.001	16 (12-23)
Lymphocytes (×10^9^/L) M(IQR)	1.04 (0.75-1.52)	1.33 (1.05-1.77)	0.002	0.9 (0.67-1.29)	1.11(0.8-1.39)	0.079	1.13 (0.79-1.49)
LDH (U/L), M(IQR)	221 (183-288)	213 (189-247)	0.471	255 (215-338)	245 (204-334)	0.675	229 (193-290)

Tα1, thymosin alpha-1; URT, upper respiratory tract; IVIG, intravenous immunoglobulin; IFNα2b, interferonα2b; LDH, lactic dehydrogenase.

### Effect of Tα1 on the Restore of CD4+ and CD8+ T Cell Counts

**Figure 1:**
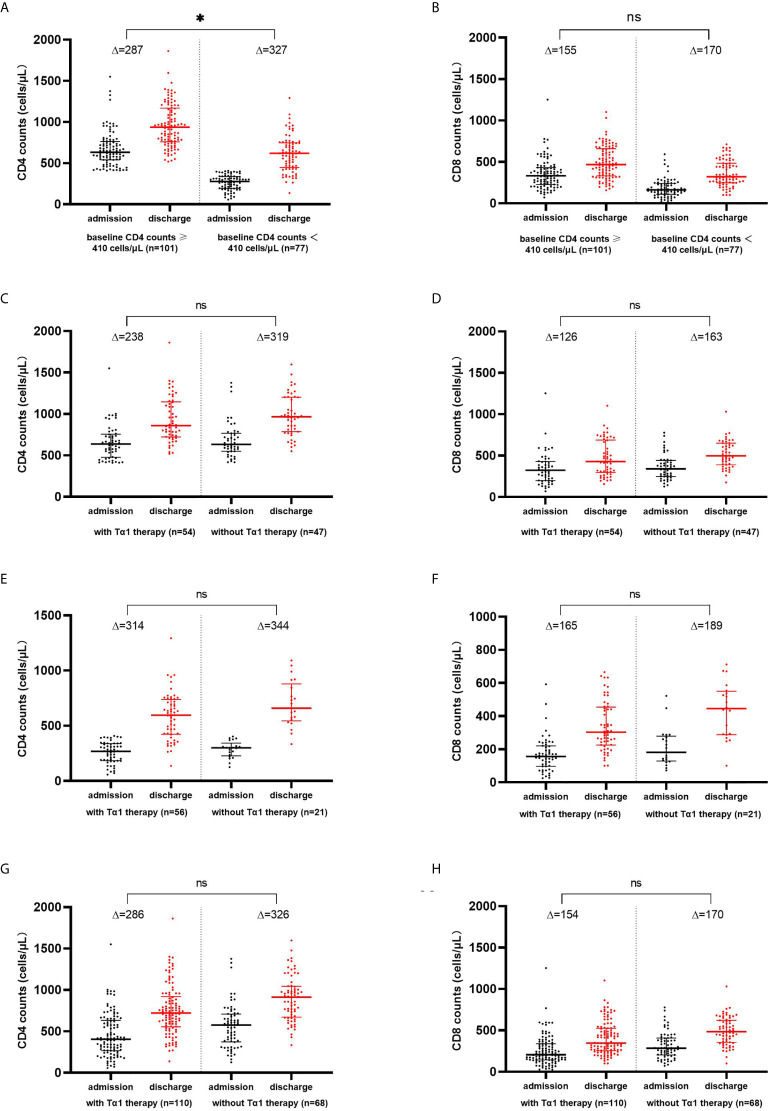
CD4+ and CD8+ T-lymphocytes counts increased from baseline during the convalescence of COVID-19. Changes of CD4+ and CD8+ T cell counts in COVID-19 patients with baseline CD4 counts ≥ 410 cells/μL and<410 cells/μL **(A, B)**, with normal baseline CD4 counts **(C, D)**, with decreased baseline CD4 counts **(E, F)**, and with or without Tα1 therapy **(G, H)**. * p<0.05; ns, no significance.

Reassessment of the lymphocyte subset profile during the convalescence period before discharge was done in 178 patients, among whom 110 received Tα1 therapy and 68 did not. Alterations in the levels of CD4+ and CD8+ T cells in the recovery period were analyzed. There was no statistical difference in the increase in CD4+ (p=0.851) or CD8+ T cell counts (p=0.842) between the two groups of patients with (n=110) or without Tα1 therapy (n=68). This was also true for the subgroups of patients with normal or decreased baseline CD4+ T cell counts. Whether or not having received Tα1 therapy, the elevation in the CD4+ T cell counts was more pronounced in the patients with decreased baseline CD4+ T cell counts (n=77) compared with those with normal baseline CD4+ T cell counts (n=101) (p=0.038); however, there was no significant difference in the increase of CD8 counts (p=0.058). See **Figure 1**.

### Factors Associated With SARS-CoV-2 Clearance in COVID-19 Patients

Multivariate linear stepwise regression analysis, which included age, gender, severity of disease, Tα1 treatment, use of glucocorticoid or IVIG, was conducted in 275 patients. The results showed that the severity of disease (p<0.001) and treatment with Tα1 (p=0.001) were correlated with duration of SARS-CoV-2 RNA detection in the throat swabs from COVID-19 patients. Nevertheless, age, gender, and use of glucocorticoid or IVIG were not correlated with the duration. See **Table 3**.

**Table 3:** Multivariate analysis of factors correlated with the duration of virus shedding in the upper respiratory tract.

Population	Factors	B	t	p
All the 275 included patients	Severity of disease	0.260	4.438	<0.001
	Tα1 treatment	-1.193	-3.305	0.001
126 patients with Tα1 therapy	Starting time of Tα1 therapy	0.556	8.612	<0.001
	Total doses of Tα1	0.403	5.094	<0.001
	Severity of disease	0.161	2.016	0.046

Tα1, thymosin alpha-1.

Of the 126 patients treated with Tα1, the median starting time of Tα1 therapy was 10 days (IQR:6-15; range:1-35) after the onset of disease, and the median number of injections was 5 (IQR: 3-7; range: 1-24). Considering the doses and starting time of Tα1 treatment varied among patients, we further conducted multivariate linear stepwise regression to clarify the effect of the two factors on the virus clearance. The other factors included in the analysis were age, gender, severity of disease, use of glucocorticoid or IVIG. As shown in **Table 3**, the time of administering Tα1 (p<0.001), the total number of doses of Tα1 (p<0.001), and severity of disease (p=0.046) were all associated with the duration of SARS-CoV-2 shedding in the upper respiratory tract.

## Discussion

According to this study, T-lymphocytes subsets changes during the course of COVID-19. The decline of CD4+ and CD8+ T cell counts was observed at the onset of disease and increased during the convalescent stage. The increases in CD4+ and CD8+ T cell counts were more pronounced in patients with decreased baseline CD4+ T cell counts, compared with those with normal baseline CD4+ T cell counts. We found that the use of Tα1 had no effect on promoting the recovery of CD4+ and CD8+ T cell counts. On the contrary, multivariate linear regression analysis revealed that the severity of the disease and use of Tα1 may actually be related with a prolonged time of virus clearance.

According to Chinese guidelines for diagnosis and treatment of COVID-19, the severity of COVID- 19 is classified as mild, moderate, severe and critical (5). It is clear that there are also, some asymptomatic persons who have been infected with SARS-CoV-2 (24). The heterogeneity of clinical manifestations of COVID-19 may be related to a variety of factors. Here we have described the clinical features of COVID-19 from the perspective of the patient’s cellular immunity status and found that patients with reduced baseline CD4+ T cell counts were more likely to be older, have higher levels of LDH, more severe symptoms, and have hypertension. Older age, hypertension, and high LDH level were all found to be risk factors for severe illness or death (25). These results suggest that lower CD4+ T cell counts may be an indicator of more severe disease, and patients should be monitored more carefully. However, we found no significant differences in the clearance time of virus and duration of hospitalization between the two groups. This suggests that changes in T-lymphocyte subsets may be correlated with the severity of the disease, but not with the duration of the disease. Qin et al. also found that the number of T lymphocytes decreased significantly in severe COVID-19 patients, and the level of both helper and inhibitory T cells were below normal in the COVID-19 group (20). Therefore, monitoring of T-lymphocyte subsets is helpful for the early screening of critical diseases, but individuals infected with SARS-CoV-2 should be managed as an equally important source of infection regardless of the patient’s clinical characteristics and immune status.

We observed that the number of CD4+ and CD8+ T cells increased during the convalescence period of COVID-19, and the increased number of CD4+ T cells in patients with low baseline CD4+ T cell counts was significantly higher than in those with normal CD4+ T cell counts. However, the mechanism of this change is unclear. The study published by Liu et al. also showed the similar results that Tα1 treatment effectively restored T-cell numbers in cases with counts of CD8+ or CD4+ T cells less than 400/μL or 650/μL, respectively. Patients with higher T-cell numbers gained no benefits from Tα1 treatment (26). However, the patients included in the two studies were different. The study published by Liu et al. included only severe and critical COVID-19 patients, whereas our study included patients mainly classified as mild or moderate types. Liu et al. investigated whether Tα1 can restore T-cell numbers in COVID-19 patients with severe lymphocytopenia by the means of comparing the changes in CD4+ and CD8+ cell counts before and after Tα1 treatment in 34 cases. However, this comparison was only self-contrast before and after Tα1 treatment other than comparisons between groups (Tα1 treatment group *vs.* Tα1 non-treatment group). It was not clear whether the increase of T-cell counts was part of the disease course or the effect of T α1 therapy. Our study also observed the increase of CD4+ and CD8+ T cell counts in the recovery period of disease in both groups of patients with and without Tα1 therapy. However, there was no significant difference in the changes of CD4+ or CD8+ T cell counts between the two groups. A multicenter retrospective study evaluated the efficacy of Tα1 for critical COVID-19 patients, and the results suggested that treatment with Tα1 could markedly decrease 28-day mortality and attenuate acute lung injury in critical type of COVID-19 patients (27). However, another study which also enrolled the critical COVID-19 patients came to the opposite conclusion - there was no association between use of thymosin α1 and decreased mortality in critically ill COVID-19 patients (28). The reasons for the different results are unknown, one study found that gender differences may be a factor in sustaining COVID-19 immunity responded to Tα1 (29).

As an immunomodulatory agent, Tα1 has shown good efficacy in a variety of diseases, including hepatitis B, cancer, sepsis, cystic fibrosis, etc. (18, 19). However, Tα1 showed no benefit for COVID-19 in our study in the perspective of restoring CD4 and CD8 counts. On the contrary, the duration of SARS-CoV-2 RNA shedding in the upper respiratory tract in patients given Tα1 therapy was significantly longer than that in those who did not. A possible explanation of the finding is that the severe and critical COVID-19 disease was more common in the Tα1 therapy group. Multivariate regression analysis showed that the severity of disease was an influencing factor of virus clearance. The more severe disease was, the longer patients shed virus.

At the present time, Tα1 is mainly used for the treatment of diseases with immunodeficiency or immune disorders. However, the physiological basal levels of Tα1 and the most effective dose and schedule of treatment remain unclear. According to expert opinion, low serum Ta1 levels are predictive and/or associated with different pathological conditions. In the case of Tα1 treatment, it is crucial to know the patient’s baseline serum Tα1 level to establish effective treatment protocols and monitor its effectiveness over time (30). COVID-19 is an emerging infectious disease, and it remains unclear just what the host immune response to SARS-CoV-2 infection is. The cytokine release syndrome (CRS) appears to affect patients with severe disease. Strategies for choosing whether to enhance or suppress the immune response may be based on the stage of and pattern of the immune reaction induced by SARS-CoV-2 virus (31). In vitro studies have shown that Tα1 could mitigate cytokine expression and inhibit lymphocyte activation in a CD8+ T-cell subset from COVID-19 patients, which suggest that the potential role of Tα1 in modulating the immune response homeostasis and the cytokine storm *in vivo* (32). It’s important to know the profiles of Tα1 and cytokines secretion during the course of COVID-19 disease, which would be a prerequisite for determining whether or not Tα1 is a useful treatment for COVID-19.

There are, of course, some limitations of this study. They would include possible selection bias, the small sample size, incomplete patient data, a retrospective study, etc. In addition, the treatment regimen of Tα1 was not standardized and may not have been a correct one. Besides, there were many influencing factors, which may affect the conclusion of statistical analysis.

In conclusion, we have demonstrated that there is an alteration in the T-lymphocyte subsets during the course of COVID-19. Tα1 appeared to have no beneficial effect neither on the recovery of CD4+ and CD8+ T cell counts nor on the virus clearance during the convalescence stage of COVID-19. We suggest that any further use of Tα1 for COVID-19 needs to be further investigated.

## Data Availability Statement

The raw data supporting the conclusions of this article will be made available by the authors, without undue reservation.

## Ethics Statement

The studies involving human participants were reviewed and approved by Ethics Committee of the Shanghai Public Health Clinical Center. Written informed consent for participation was not required for this study in accordance with the national legislation and the institutional requirements.

## Author Contributions

All authors contributed to the article and approved the submitted version. HL have contributed to the conception and design. ZW, JC, and CZ were in charge of writing, data collection and analysis. LL, TQ, YS, YZ, LX, TL, and ZQ contributed to the data collection. CS revised the manuscript.

## Funding

This work was funded by the Fudan university (IDF162005), Shanghai Public Health Clinical Center (2020YJKY01), Shanghai major projects on infectious diseases (shslczdzk01102), Shanghai Municipal Health Commission Scientific Research Project (201940225), Shanghai Science and Technology Commission (20411950200), and Chinese medicine administration of Shanghai Health Protection Committee (2020NCP001).

## Conflict of Interest

The authors declare that the research was conducted in the absence of any commercial or financial relationships that could be construed as a potential conflict of interest.
